# Retinoid‐induced skeletal hyperostosis in disorders of keratinization

**DOI:** 10.1111/ced.15382

**Published:** 2022-09-27

**Authors:** Brent J. Doolan, Alexandra Paolino, Danielle T. Greenblatt, Jemima E. Mellerio

**Affiliations:** ^1^ St John's Institute of Dermatology Guy's and St Thomas' NHS Foundation Trust London UK; ^2^ St John's Institute of Dermatology, School of Basic and Medical Biosciences King's College London UK

## Abstract

For disorders of keratinization, topical treatment alone may be ineffective, and systemic retinoid therapy may be indicated. Treatment with systemic retinoids (acitretin, isotretinoin and alitretinoin) has been shown to be effective in reducing disease severity; however, potentially rare adverse effects (AEs) may occur, including hyperostotic skeletal changes. The true prevalence of this AE in adult patients administered life‐long therapy is unknown. We identified 3 of 127 (2.4%) patients (with ichthyosis or Darier disease) who had been prescribed isotretinoin with or without acitretin, and who developed radiological signs and clinical symptoms of hyperostosis and ligamentous ossification. This clinical review highlights the significance of retinoid‐induced skeletal hyperostosis in patients prescribed long‐term, high‐dose retinoid therapy for disorders of keratinization. Patients commencing systemic retinoid therapy, particularly women of childbearing age, should be counselled about this important and potentially serious AE, especially if long‐term treatment is indicated.

## Graphical abstract

Systemic retinoid therapy may be associated with potentially rare adverse effects (AEs) such as skeletal hyperostosis. This paper highlights the significance of retinoid‐induced skeletal hyperostosis in patients prescribed long‐term, high‐dose retinoid therapy for disorders of keratinization. Patients commencing systemic retinoid therapy, particularly women of childbearing age, should be counselled of this important and potentially serious AE, especially if long‐term treatment is indicated.

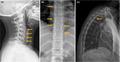

Disorders of keratinization encompass a number of genetic skin diseases that share abnormalities in epidermal differentiation, often with aberrant manufacturing of corneocytes.^1^ For many of these disorders, topical treatment alone may be ineffective, and systemic retinoids such as acitretin, isotretinoin and alitretinoin may be indicated to reduce disease severity. These drugs, metabolites of vitamin A, have been shown to significantly reduce scale and hyperkeratosis, while improving patient quality of life.^2^ However, data from clinical and observational studies (psoriasis, acne vulgaris and paediatric ichthyosis) have noted an association with potentially significant rare or very rare adverse effects (AEs) such as hyperostotic changes involving the tendons and ligaments of the spine and joints (osteophytes, calcification of tendons and ligaments, and hyperostosis).^2^, ^3^, ^4^ Given the systemic dosing required to treat disorders of keratinization, as well as the possibly life‐long duration of therapy, investigation into the significance and true prevalence of this AE in this specific cohort is warranted.

A retrospective review was undertaken of patients prescribed systemic retinoids, who attended the adult genodermatoses clinic at our centre between July 2008 and April 2022. Data were collected on demographics, diagnosis, systemic retinoid therapy used, treatment duration, total cumulative dose and skeletal system AEs.

Over the study period, 127 of 566 patients with disorders of keratinization had been or were currently being treated with systemic retinoids (Table 1). Retinoid therapy was most frequently used for ichthyosis (51 of 127; 38.6%), palmoplantar keratoderma (32 of 127; 25.2%) and Darier disease (27 of 127; 21.3%). Acitretin was prescribed most frequently, with isotretinoin prescribed predominantly for female patients and for a shorter duration, compared with acitretin and alitretinoin. We noted a younger age for commencement of isotretinoin compared with other systemic agents. The daily dose by weight was within the normal range limits for all systemic agents, and we observed high total cumulative doses for all agents, reflecting disease chronicity.

**Table 1:** Demographic and treatment information for patients with disorders of keratinization administered systemic retinoid therapy.

Characteristics	Total (*n* = 127)	Treatment groups^a^		
		Acitretin (*n* = 107)	Isotretinoin (*n* = 40)	Alitretinoin (*n* = 6)
Female sex, *n* (%)	66 (52.0)	48 (44.9)	35 (87.5)	4 (66.7)
Diseases treated				
Ichthyosis	51 (38.6)	42 (39.3)	18 (45.0)	3 (50.0)
Palmoplantar keratoderma	32 (25.2)	28 (26.2)	7 (17.5)	3 (50.0)
Darier disease	27 (21.3)	24 (22.4)	8 (20.0)	0 (0)
Pachyonychia congenita	5 (3.9)	5 (4.7)	0 (0)	0 (0)
Hailey–Hailey disease	4 (3.1)	3 (2.8)	1 (2.5)	0 (0)
Other^b^	8 (6.3)	5 (4.7)	6 (15.0)	0 (0)
Age at commencement, years^c^ ^,^ ^d^	31.9 ± 17.1	32.1 ± 17.3	27 (19;33)	35 (29; 38)
Total cumulative dose, mg, median (Q1, Q3)^d^	–	45 734.5 (16 425; 109 500)	21 900 (7300; 73 000)	27 375 (17 793.8; 32 850.0)
Daily dose, mg/kg/day, mean ± SD	–	0.33 ± 0.22	0.36 ± 0.22	NA
Time on treatment, years^c^ ^,^ ^d^	11.2 ± 110.0	10.3 ± 110.1	2.9 (1; 7)	3 (1.9; 5.3)
Ossification, *n* (%)	3 (2.4)	1 (0.9)	3 (7.9)	0 (0)

NA, not applicable; Q, quartile.

^a^ Treatment groups were not mutually exclusive.

^b^ Included acrokeratoelastoidosis, erythrokeratodermia variabilis, Papillon–Lefèvre syndrome, keratosis follicularis spinulosa keratosis, follicularis spinulosa decalvans, keratosis lichenoides chronica and lipoid proteinosis.

^c^ Mean ± SD or median (Q1, Q3).

^d^ Median (Q1, Q3) were used for data that were not normally distributed.

Overall, we identified 3 of 127 (2.4%) patients (with ichthyosis or Darier disease) who had been prescribed isotretinoin with or without acitretin, who developed radiological signs of hyperostosis and ligamentous ossification (Fig. 1). In addition, all three developed clinical symptoms of joint stiffness, as well as discomfort and reduced flexibility when bending over and/or transferring (Supplementary Table S1). All three patients had systemic treatment discontinued and were referred to rheumatology services for further investigation and to exclude other differential diagnoses, notably ankylosing spondylosis.

**Figure 1:**
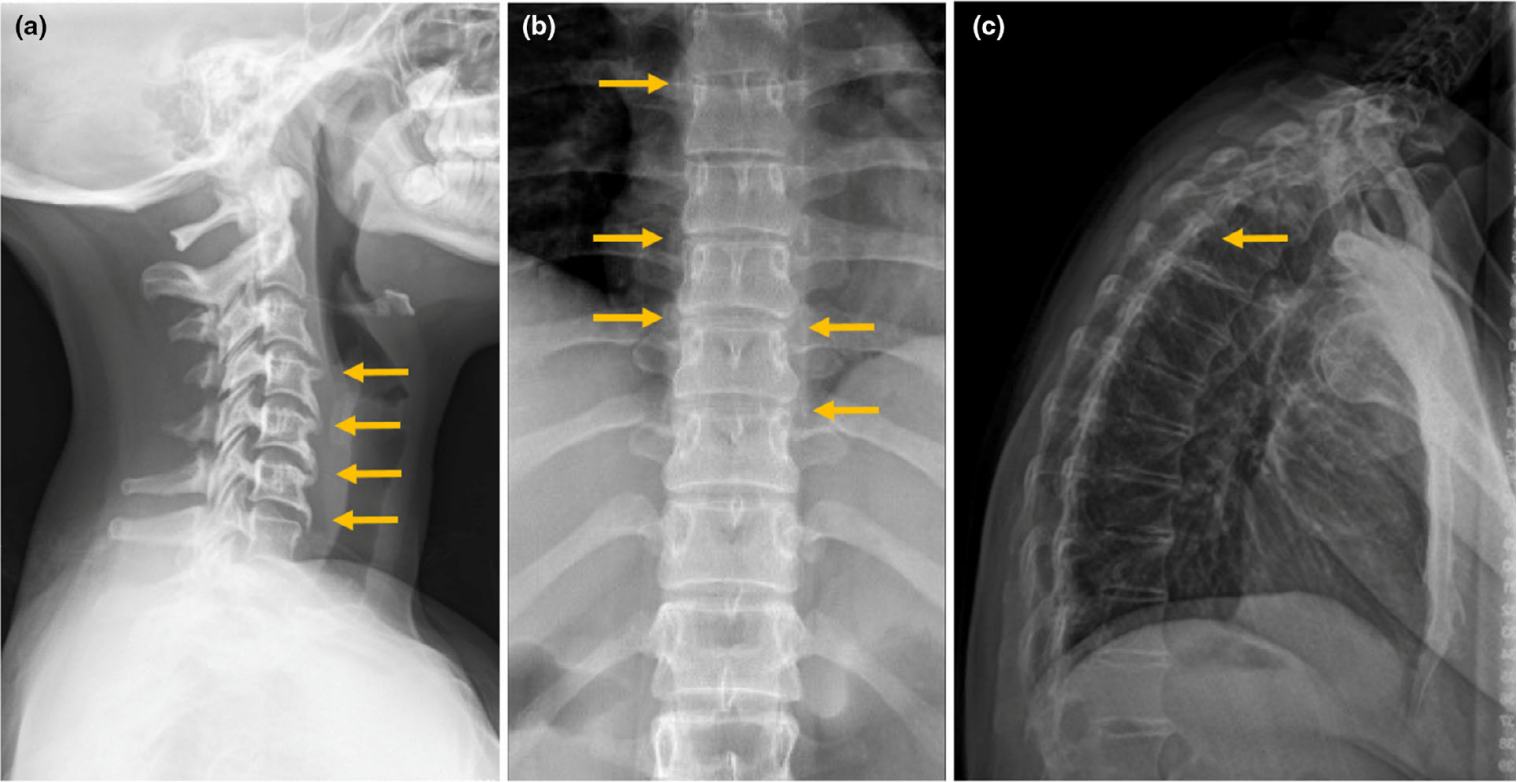
(a) A lateral cervical spine radiograph of a 25‐year‐old woman (Patient 1) who was treated with acitretin and isotretinoin for lamellar ichthyosis, showing radiographic features of straightening of the normal cervical lordosis and anterior ossification from C3 to C7 (orange arrows), with appearances in keeping with skeletal hyperostosis. (b) An anterior thoracic spine radiograph of a 38‐year‐old woman (Patient 2) who was treated with isotretinoin for Darier disease, showing radiographic features of bridging osteophytes seen bilaterally (orange arrows) and anteriorly through the thoracic spine. (c) A lateral thoracolumbar spine radiograph of a 43‐year‐old woman (Patient 3) who was treated with isotretinoin for epidermolytic ichthyosis, with radiographic features showing diffuse syndesmophytosis with ossification of the anterior longitudinal ligament, with a linear longitudinal radiodense stripe also running centrally along the vertebral column (yellow arrow) in keeping with ossification of the interspinous/supraspinous ligaments with loss of the normal lumbar lordosis and in keeping with diffuse hyperostosis.

This clinical review highlights the significance and high prevalence of retinoid‐induced skeletal hyperostosis in patients prescribed long‐term, high‐dose retinoid therapy for disorders of keratinization. Disease progression does not continue after cessation of retinoid therapy, as a follow‐up radiological study noted no further hyperostotic vertebral abnormalities ~16 months after stopping treatment.^5^ However, the development of clinically significant skeletal symptoms is permanent, with management focusing on cessation of systemic retinoids, maintaining and preserving flexibility with hydrotherapy and physiotherapy, treating stiffness with simple analgesia, and managing associated risk factors including gout, hyperlipidaemia and diabetes.^6^ Our data reflect the dosing regimen and AE profile of patients over the allocated time period, but data on retinoid use prior to 2008 were not available for many patients. This limitation underestimates the total cumulative dosing of those with congenital disease, but still allows for an accurate assessment of AEs.

It is not known whether the observed retinoid‐induced skeletal toxicity in these patients is due to specific drug–receptor involvement, to disease severity or to the dosage and frequency of treatment, but involvement of isotretinoin in all cases highlights a predilection for drug‐specific skeletal‐disease development.^7^ For all three cases, their total cumulative dosing was high (Supplementary Table S1), putting them at an increased risk of toxicity.^7^ This is of particular importance as all cases were in women who required the use of isotretinoin during their childbearing years, in order to avoid the 3‐year washout for acitretin that is recommended prior to conception.^7^ It must also be noted that hyperostotic bony changes are extremely common in the general population and increase with age even without exposure to retinoid treatment.^2^ One study found that 100% of 400 spine specimens of individuals > 40 years of age showed some degree of anterior osteophyte formation; however, all these cases were asymptomatic.^8^

Monitoring for skeletal‐related issues should include taking a personal and family medical history of any significant musculoskeletal problems, as well as a regular inquiry about musculoskeletal symptoms. If symptoms or personal/family medical history suggest musculoskeletal or joint pathology, then radiographic investigation is suggested. If a patient reports aches or stiffness during treatment, radiographic investigations of the symptomatic areas can help characterize skeletal disease. Radiography should include lateral views of the cervical, thoracic, and sometimes lumbar spine.^2^ Patients commencing systemic retinoid therapy, particularly women of childbearing age, should be counselled of this important and potentially serious AE, especially if long‐term treatment is indicated.

**Learning points**

- Treatment with systemic retinoid therapy may be indicated for effective treatment of disorders of keratinization.
- Potentially rare AEs such as skeletal hyperostosis and ligament ossification may occur with the use of systemic retinoid therapy, particularly in patients prescribed high‐dose, life‐long therapy.
- Monitoring for skeletal‐related issues should include taking a personal and family medical history of any significant musculoskeletal problems at screening, and following up with regular enquiry about such problems during treatment.
- If a patient reports aches or stiffness at screening or during treatment, radiographic investigations of the symptomatic areas can help characterize skeletal disease.

## Conflict of interest

The authors declare that they have no conflict of interest.

## Funding

None.

## Ethics statement

Ethics approval by the institutional review board was not required; approval was granted from the Department of Dermatology, Guy's and St Thomas' NHS Foundation Trust. Patients provided informed consent for publication of their case details and images.

## Supporting information


**Supplementary Table S1** Analysis of patients who developed retinoid‐induced skeletal changes.

## Data Availability

Data are available on request from the corresponding author.
