# Supplementary material for: Retinoid‐induced skeletal hyperostosis in disorders of keratinization
Source: Clin Exp Dermatol. 2022 Sep 27;47(12):2273–6. doi: 10.1111/ced.15382 (PMC10087317; doi:10.1111/ced.15382)
Supplement: Supplementary file 1 — Supplementary Table S1 Analysis of patients who developed retinoid‐induced skeletal changes. [file CED-47-2273-s001.docx]

**Retinoid-induced skeletal hyperostosis in disorders of keratinisation**

Doolan BJ, Paolino A, Greenblatt DT, Mellerio JE

**______________________________________________________________________________________________________________________________**

**Supplementary File:**

**Table S1:** Analysis of cases that developed retinoid-induced skeletal changes.

| **Characteristics** | **Case 1** | **Case 2** | **Case 3** |
| --- | --- | --- | --- |
| Indication for systemic retinoid therapy | Lamellar ichthyosis | Darier Disease | Epidermolytic ichthyosis |
| Age at systemic retinoid commencement (years) | 1 | 14 | 11 |
| *Time on treatment (years)* |  |  |  |
| Isotretinoin | 8 | 21 | 18 |
| Acitretin | 13 | - | - |
| *Total cumulative dose (mg)* |  |  |  |
| Isotretinoin | 131,400 | 405,150 | 255,500 |
| Acitretin | 106,763 | - | - |
| Past medical history | Collodion membrane in infancy | Vitamin D deficiency | Onychomycosis |
| Other long-term systemic medications | Cholecalciferol | Microgynon (ethinylestradiol and levonorgestrel) Cholecalciferol | Microgynon (ethinylestradiol and levonorgestrel) |
| Family history of joint pathology | No | No | No |
| Presenting symptoms | Progressive stiffness in the lower back, shoulders, neck and hips Discomfort when walking Worsening flexibility Decreased plantar flexion | Worsening chronic back pain Generalised stiffness Decreased range of movement | Progressive neck stiffness Unable to raise her arms Unable to straighten arms: completely rigid Right hip: fixed flexion |
| Practical issues | Difficulty putting on shoes Cannot cross legs Feet constantly ache Shoulder discomfort affecting sleep | Mild difficulty transferring out of bed (morning worse) | Neck stiffness affecting sleep Difficulty reaching items in top cupboards General ache when sitting at work desk |
| Radiological findings | Widespread severe ossifying enthesopathy affecting peripheral and axial skeleton. | Diffuse Idiopathic Skeletal Hyperostosis-like pathology Significant disc osteophyte protrusion at cervical spine | Ossification of the interspinous/ supraspinous ligaments Ossification extending from the transverse processes to the iliac crests bilaterally.  Diffuse bony spurs: pelvic brim, ASIS, ischial tuberosities, and lesser trochanters bilaterally, & greater trochanter. |
| ASIS = Anterior superior iliac spine |  |  |  |
